# Practice intentions at entry to and exit from medical schools aspiring to social accountability: findings from the Training for Health Equity Network Graduate Outcome Study

**DOI:** 10.1186/s12909-018-1360-6

**Published:** 2018-11-13

**Authors:** Sarah Larkins, Karen Johnston, John C. Hogenbirk, Sara Willems, Salwa Elsanousi, Marykutty Mammen, Kaatje Van Roy, Jehu Iputo, Fortunato L. Cristobal, Jennene Greenhill, Charlie Labarda, Andre-Jacques Neusy

**Affiliations:** 1 College of Medicine and Dentistry, James Cook University, Townsville, Queensland Australia; 2 Anton Breinl Research Centre for Health Systems Strengthening, James Cook University, Townsville, Queensland Australia; 3 Training for Health Equity Network, New York, NY USA; 4 Centre for Rural and Northern Health Research, Laurentian University, Sudbury, Ontario Canada; 5 Department of Family Medicine and Primary Health Care, Ghent University, Ghent, Belgium; 6 Community Medicine, Department of Family and Community Medicine, University of Gezira, Gezira, Sudan; 7 Teaching and Learning Centre, University of Fort Hare, East London, Eastern Cape South Africa; 8 Department of Medical Education, Walter Sisulu University, Mthatha, South Africa; 9 School of Medicine, Ateneo de Zamboanga University, Zamboanga City, Philippines; 10 School of Medicine, Flinders University, Adelaide, South Australia; 11 School of Health Sciences at the University of the Philippines, Manila, Philippines; 12 James Cook Drive, James Cook University, Townsville, 4810 Australia

**Keywords:** Medical education, Health workforce, Social accountability, Learner characteristics, Practice intention

## Abstract

**Background:**

Understanding the impact of selection and medical education on practice intentions and eventual practice is an essential component of training a fit-for-purpose health workforce distributed according to population need. Existing evidence comes largely from high-income settings and neglects contextual factors. This paper describes the practice intentions of entry and exit cohorts of medical students across low and high income settings and the correlation of student characteristics with these intentions.

**Methods:**

The Training for Health Equity Network (THEnet) Graduate Outcome Study (GOS) is an international prospective cohort study tracking learners throughout training and ten years into practice as part of the longitudinal impact assessment described in THEnet’s Evaluation Framework. THEnet is an international community of practice of twelve medical schools with a social accountability mandate. Data presented here include cross-sectional entry and exit data obtained from different cohorts of medical students involving eight medical schools in six countries and five continents. Binary logistic regression was used to create adjusted odds ratios for associations with practice intent.

**Results:**

Findings from 3346 learners from eight THEnet medical schools in 6 countries collected between 2012 and 2016 are presented. A high proportion of study respondents at these schools come from rural and disadvantaged backgrounds and these respondents are more likely than others to express an intention to work in underserved locations after graduation at both entry and exit from medical school. After adjusting for confounding factors, rural and low income background and regional location of medical school were the most important predictors of intent to practice in a rural location. For schools in the Philippines and Africa, intention to emigrate was more likely for respondents from high income and urban backgrounds.

**Conclusions:**

These findings, from a diverse range of schools with social accountability mandates in different settings, provide preliminary evidence for the selection and training of a medical workforce motivated to meet the needs of underserved populations. These respondents are being followed longitudinally to determine the degree to which these intentions translate into actual practice.

## Background

Globally, shortages and maldistribution of the health workforce (often termed Human Resources for Health) impede the strengthening of primary health care focussed health systems and improvement in health outcomes [1]. These issues are particularly acute for those in low and middle-income countries (LMICs). A mal-distributed health workforce contributes to failures to achieve Universal Health Coverage (UHC) in all countries [2]. Simply adding more qualified health workers into the mix is likely to have little impact, without linking health professional education (HPE) to the needs of local health systems and thus addressing issues of distribution as well [3]. These pressing issues have led the World Health Organization (WHO) and other global health organisations to focus on broadening health professional education to recruit students from underserved areas, train them mostly in the community with a primary care focussed curriculum and monitor graduate outcomes – the transformative HPE agenda [1].

Increasingly, medical educators recognize that not only should medical school graduates be competent physicians, they should also be equipped to meet the challenges of providing care to underserved populations through a motivation to work with, and in, underserved communities. These changes and additions to desired graduate attributes are gradually being reflected in national and international medical school accreditation frameworks [4, 5]. In response to these challenges, several health professional schools have had strong social missions for some time. Many of these schools are relatively small and poorly resourced, thus these medical education programs and the often impressive outcomes of these schools have had limited impact on a global stage [6]. While there is good evidence on the effectiveness of preparation for rural practice in Australia and Canada, [7] little is understood globally about the interactions and impacts of what happens within the schools that might influence intentions to practice and actual practice [8, 9].

The Training for Health Equity Network (THEnet), formed in 2008, is a community of practice of twelve medical schools with an explicit social accountability mandate. These schools are located in both high and low income settings around the world and share a mission to: 1) meet the priority health needs of the population that they serve, with a particular focus on the needs of underserved populations; and 2) produce graduates with the knowledge, attitudes and skills to meet these needs [10]. The way in which these schools operationalize this shared mission varies in response to their context, but certain common features have been previously reported [10].

THEnet’s first piece of collaborative work was to develop a common Evaluation Framework for measuring progress towards social accountability [11]. To evaluate outcomes and impact we then developed tools and processes for an international graduate outcome study involving THEnet schools in a range of contexts [12]. This prospective cohort study involves surveys with entering medical students at THEnet schools, who are surveyed again at exit from medical school and at postgraduate Years 1, 4, 7 and finally 10 years after graduation. This will enable us to develop an international picture of both intended and actual practice discipline and location as well as other practice characteristics (e.g. populations served, scope of practice) in a broader range of settings than is previously available [12].

In high income settings, practice intention at entry and graduation has been shown to correlate with actual practice location, both in early postgraduate years and in subsequent practice [13, 14]. However, some students change their intentions [8] and another question that needs to be answered is what happens to practice intention between entry to and exit from medical schools aspiring to social accountability. This is a necessary first step to beginning to understand the potential mediators of these changes, such as curricula, placement experiences, exposure to role models and so on. Additionally, it is vital to understand how these potential mediators might affect the intentions of students from different backgrounds and in different countries, in order to expand these strategies to a broader range of schools. This manuscript aims to: 1) describe characteristics associated with intention to practice with underserved populations for entry and exit cohorts at THEnet medical schools, and; 2) consider student characteristics associated with differences in practice intention across different contexts.

## Methods

This is a multi-site, prospective study involving eight medical schools in six countries and five continents (Table 1). The THEnet Graduate Outcome Study (GOS) is an international cohort study tracking learners throughout training and ten years into practice as part of the longitudinal impact assessment described in THEnet’s Evaluation Framework [11, 12]. Data presented here include cross-sectional entry and exit data obtained from different cohorts of medical students.

**Table 1:** Context for THEnet schools participating in the THEnet Graduate Outcome Study

School	Priority population	Graduate entry (Training route^a^)	Length of training (Years)	Program	Time of applying for specialty training
Ateneo de Zamboanga University School of Medicine (ADZU) Zamboanga city, Mindanao	Rural underserved areas of Mindanao, Philippines	Yes (VI)	4	50% community based	After PGY1^b^
Flinders University School of Medicine (FU) Adelaide, Australia	Rural, remote, Aboriginal and Torres Strait Islander populations.	Yes (VI)	4	Parallel Rural Community Curriculum (1 year, 30 students) Northern Territory Clinical School (6 month remote clinical placement, 8 students) Short rural placements in Year 1 Cultural Awareness training 3rd year options of Rural LIC (40 weeks) Or Rural/Urban LIC (20/20 weeks) Or NT Darwin/Remote program (20/20) LIC. 4th year-rural/remote & overseas electives. Student societies with SA focus.	After PGY1
University of Gezira Faculty of Medicine Gezira State, Sudan	Rural underserved areas in Gezira	No (IV)	5	25% curriculum community based, community oriented education	After PGY1
Ghent University Ghent, Belgium	Low socio-economic status, migrant population	No (I)	7	6-year learning continuum on ethnic, gender and socio-economic diversity: basic competence training and 6 weeks course on social determinants of health (Y1), a one week community oriented primary care program (Y2), specialist courses on social determinants of health and diversity, further competency training, and several community/primary care based clinical internships (Y3–6).	End of year 6
James Cook University (JCU) Townsville, Australia	Rural, remote, Aboriginal and Torres Strait Islander populations.	No (VI)	6	Entire program located in outer regional and rural settings with focus on priority health needs, 20 weeks training in rural and remote settings Year 4–6 based in health care facilities	After PGY1
Northern Ontario School of Medicine (NOSM) Thunder Bay and Sudbury, Canada	Rural, Indigenous, Francophone and general populations of Northern Ontario.	Yes (V)	4	Year 1 and 2: 4 weeks and 8 weeks in Indigenous and rural communities, Year 3 based in rural family practice setting (8 month comprehensive community (longitudinal integrated) clerkship)	Beginning of Year 4 of medical course (8 months prior to graduation)
University of the Philippines School of Health Sciences (UPSHS) Leyte, Philippines	Rural underserved areas in the Philippines; Indigenous people groups	Yes (VI)	5	Multi-level entry stepladder curriculum Year 2: 6 months rural community placement Year 5 based in municipal health community practice setting	PGY1
Walter Sisulu University Faculty of Health Sciences (WSU) Mthatha, South Africa	Rural underserved areas of Eastern Cape and KwaZulu Natal Provinces of South Africa	No (IV)	6	Rural experiences in Years 1–3 Year 1: 6 days per year; Year 2: 3 weeks per year; Year 3: once a week from February to October. i.e., once weekly 36 weeks/year. 6 months rural placement in Year 5	Following 2 year internship and subsequent 1 year of community service. (After PGY3)

^a^Pathway classification for medical education[31]

^b^PGY1 Postgraduate year one

All learners entering and graduating from these medical schools were invited to complete the GOS questionnaires. The questionnaires assessed learners’ socio-economic and demographic background characteristics, rurality of primary schooling, choice of medical school and practice intentions (location, discipline of practice and populations to be served). As reported previously, questionnaires were created based on the widely used Australian Medical Students Outcomes Database (MSOD) Medical Students’ Questionnaires [15] and modified through discussions held among THEnet partner schools to create variables that can be consistently applied across contexts [12]. These discussions explored the dimensions of disadvantage of populations served by each school. Disadvantage was assessed based on geography, parental family income and sociocultural disadvantage, adapted to each school’s context [16]. The modified instruments were reviewed by medical education leaders from THEnet schools for both content and construct validity, and piloted at a high-income and a low-income school, resulting in minor revisions in wording [17].

At each school, incoming students were invited to complete the entry questionnaire in the first months of their medical education program over 2012 to 2016 in either paper or electronic format. The exit questionnaires were administered to different cohorts of students exiting the course in the last few months of their medical program. There has been a staggered commencement of the project as schools completed their individual ethics requirements.

The surveys used in different schools were identical, with the exception of variations of the descriptors for quintiles for socio-economic status and rurality, which were developed with the assistance of local experts from each country. One school, School of Medicine at Ghent University, translated the surveys into Flemish, using standard methods of translation and back-translation to assess fidelity of translation. Each school used the same codebook, and data files were merged into a Microsoft Excel file for cleaning and then analysed using SPSS Statistics for Windows Version 20 (IBM; Armonk, NY). Approval for the study was obtained from the ethics committees at James Cook University, Ghent University Hospital, Laurentian and Lakehead Universities and Walter Sisulu University and from senior academic leadership at the other schools. Informed consent was obtained from all respondents.

Data were analysed using simple frequencies and proportions, with Pearson’s chi-squared tests used to compare grouped categories, with the calculation of odds ratios, 95% confidence intervals (CI) and *p*-values to compare proportions in the entry and exit populations. Binary logistic regression was used to generate odds ratios adjusted for age, gender, student background and medical school where appropriate. Rurality of background was grouped to a dichotomous variable; quintiles 1 to 3 for rural background and quintiles 4 and 5 for urban background. Given the differences in context and underserved populations, some of the questions related to rurality of background and intention to work with rural populations were not applicable to participants from Ghent University, thus these participants were omitted for some analyses. When considering intention to practice rurally by medical school, two schools (NOSM and SHS) were excluded due to small sample sizes. The remaining five schools were dichotomized on the basis of the main medical school location into regionally-based schools (with no capital city campus; ADZU, JCU and WSU) and less regional schools (where at least some training takes place in capital cities; Flinders and Gezira). The large sample size meant that cell frequencies were not an issue for any analysis.

## Results

### Participation rates

Findings from 3346 learners from eight THEnet medical schools in six countries (five continents) are included (Table 2). Response rates overall (calculated based on total numbers of students/potential responders in the included cohorts) were 76.2% for the entry surveys and 48.6% for the exit surveys (likely due to geographical dispersion of students in their final year).

**Table 2:** Response rates for entry and exit questionnaires for THEnet schools

Medical school	Respondents (Response rate %)	
	Entry *N* = 2557/3356 (76.2%)	Exit *N* = 789/1625 (48.6%)
Ateneo de Zamboanga University, Philippines	143/146 (97.9)	76/85 (89.4)
Flinders University, Australia	218/303 (71.9)	71/145 (49.0)
Gezira University, Sudan	570/888 (64.2)	59/199 (29.6)
Ghent University, Belgium	294/462 (63.6)	165/271 (60.9)
James Cook University, Australia	736/862 (85.4)	278/654 (42.5)
Northern Ontario School of Medicine, Canada	22/64 (34.4)	8/63 (12.7)
Walter Sisulu University, South Africa	563/616 (91.4)	104/176 (59.1)
University of the Philippines, Philippines	11/15 (73.3)	28/32 (87.5)

### Demographic comparisons

Although these were different cohorts, there were no statistically significant differences between entry and exit groups in the percentage who were female and the percentage originating from a rural background, with just the expected difference in years of age given progression through the medical degree. However, relative to the exit cohort, the entry cohort had a significantly higher percentage of respondents from the lowest two socioeconomic quintiles, those who identified as a member of an underserved group, and those who reported that neither parent attended university. The entry cohort also had a significantly lower percentage of respondents with four or more years of public school between entry and exit groups (Tables 3 and 4).

**Table 3:** Demographic profile and background characteristics for all participating THEnet schools combined at entry and exit

	Mean age (SD)	Female n/N (%)	Lowest two quintiles of income (background) n/N (%)	Identify as underserved population n/N (%)	Neither parent attended university n/N (%)	Years of public schooling (> 4 years) n/N (%)	Rural background 1-3^a^ n/N (%)
Entry (*N* = 2557)	*n* = 2530 20.07 (4.005) 95% CI 19.91–20.23	1535/2556 (60.1)	475/1643 (28.9)	645/2303 (28.0)	515/2502 (20.1)	691/2250 (30.7)	825/1904 (43.3)
Exit (*N* = 789)	*n* = 755 25.25 (3.213) 95% CI 25.02–25.48	492/786 (62.6)	142/617 (23.0)	118/704 (16.8)	131/783 (16.7)	370/779 (47.5)	216/538 (40.1)
OR at entry versus exit; *p*-value for Pearson’s χ^2^ for OR	–	0.90; *p* = 0.2	1.36; *p* = 0.005	1.93; *p* < 0.001	1.29; *p* < 0.02	0.49; *p* < 0.001	1.14; *p* = 0.2

^a^Rural quintiles (1 = remote village, 2 = small rural town, 3 = large rural town) vs Urban quintiles (4 = major regional centre and 5 = major city or capital city). Respondents from Ghent University or those with primary school background in a country other than the country where they attended medical school were excluded from this variable. Most schools used population size to define quintiles; NOSM and UPSHS based quintiles on government socioeconomic classifications

**Table 4:** Demographic profile and background characteristics for respondents at each THEnet school and all participating THEnet schools combined

	Ateneo de Zamboanga University	Flinders University	Gezira University	Ghent University	James Cook University	Northern Ontario School of Medicine	University of Philippines	Walter Sisulu University	THEnet schools combined
	n/N (%)	n/N (%)	n/N (%)	n/N (%)	n/N (%)	n/N (%)	n/N (%)	n/N (%)	n/N (%)
Mean age (years; SD)	23.20; 3.086	25.71; 5.744	19.48; 2.734	21.37; 3.657	20.30; 3.809	27.53; 5.716	28.81; 4.081	20.99; 4.498	21.26; 4.413
Female^a^	144/219 (65.8)	155/289 (53.6)	360/629 (57.2)	297/458 (64.8)	634/1014 (62.5)	23/30 (76.7)	27/39 (69.2)	387/664 (58.3)	2027/3342 (60.7)
Lowest two quintiles for parent income^a^	33/160 (20.6)	64/206 (31.1)	78/385 (20.3)	21/428 (4.9)	141/589 (23.9)	3/26 (11.5)	10/34 (29.4)	267/432 (61.8)	617/2260 (27.3)
Neither parent attended university^a^	8/211 (3.7)	37/284 (13.0)	181/605 (29.9)	36/451 (8.0)	131/1009 (13.0)	4/30 (13.3)	5/38 (13.2)	244/649 (37.6)	646/3285 (19.7)
Identify as underserved group^a^	33/214 (15.4)	10/255 (3.9)	65/557 (11.7)	64/433 (14.8)	72/877 (8.2)	12/18 (66.7)	1/37 (2.7)	506/616 (82.1)	763/3007 (25.4)
Majority of primary school in remote village, small rural town, or large rural town (quintile 1–3)^b^	36/215 (16.7)	76/224 (33.9)	113/502 (22.5)	Not applicable	293/776 (37.8)	7/30 (23.3)	23/38 (60.5)	493/657 (75.0)	1041/2442 (42.6)

^a^Data were coded as missing if respondent did not give response, or chose option ‘don’t want to answer question’ or ‘unsure’

^b^Respondents from Ghent University or those with primary school background in a country other than the country where they attended medical school were excluded from this variable

Considering all respondents combined, 27.3% (617/2260) came from the lowest two quintiles of socio-economic income as judged by family income and for 19.7% (646/3285) neither parent had attended university; there was a strong association between these two variables (χ^2^ = 300.0, df = 1, *p* < 0.001). For the respondents for whom rurality of background is relevant (i.e. all except Ghent University) there was an odds ratio of 2.5 (95% CI 2.2–2.8) for THEnet respondents originating from a rural background (quintiles 1–3; 1041/2442, 42.6%) when compared with all entering medical students in Australia; the only published source of comparison data (674/2898, 23.2%) [18].

### Intended practice location

There was a strong statistically significant positive association between rural background and intended rural practice location for both entering and exiting student respondents (χ^2^ = 168.6, df = 1, *p* < 0.001; χ^2^ = 15.7, df = 1, *p* < 0.001). Other strong positive univariate associations were found with intended rural practice location for low parental income (OR 2.13) and attending a medical school situated in a regional location (OR 1.6; both *p* < 0.001; Table 5). After adjusting for the socio-demographic factors of age, gender, parental income, attending a regionally-based (rather than metropolitan-based) school and belonging to an underserved group, participants from a rural background were twice as likely to intend to practice in a rural location (with rural background and rural practice location defined as response quintiles 1–3; AOR 2.03, 95% CI1.59–2.58, *p* < 0.001; Table 5). Respondents with a low income background were 1.8 times more likely to intend to practice in a rural location (AOR 1.82, 95% CI 1.42–2.35, *p* < 0.001; Table 5) and those from a school in a regional location were 2.2 times as likely to intend rural practice (AOR 2.19, 95% CI 1.69–2.84, *p* < 0.001; Table 5). Overall, 48.5% (1032/2127) of respondents at entry reported an intention to work in a rural location, and this dropped slightly, but significantly, to 41.1% (246/599) of the exit cohort (*p* = 0.001).

**Table 5:** Predictors of intention to work in a rural location where binary variable is rural versus urban location^a^

	Number in unadjusted analysis	Unadjusted odds ratios (95% CI; *p*-value)	Adjusted odds ratios (95% CI; *p*-value) (*n* = 1287)^b^
Age	2686	1.02 (1.00–1.03; 0.071)	1.00 (o.98–1.03;NS)
Female	2724	1.29 (1.11–1.50; 0.001)	0.81 (0.64–1.02; 0.07)
Income bottom two quintiles	1752	2.13 (1.74–2.61; < 0.001)	1.82 (1.42–2.35; < 0.001)
Identify as underserved group	2442	1.92 (1.60–2.30; < 0.001)	0.92 (0.70–1.22; NS)
Rural background (Quintiles 1, 2 and 3)	2312	2.77 (2.34–3.29; < 0.001)	2.03 (1.59–2.58; < 0.001)
Attend a regionally-based medical school^c^ (ADZU, JCU and WSU)	2660	1.60 (1.50–1.17; < 0.001)	2.19 (1.69–2.84; < 0.001)

^a^Rural quintiles (1 = remote village, 2 = small rural town, 3 = large rural town) versus Urban quintiles (4 = major regional centre and 5 = major city or capital city). Excludes respondents from Ghent University

^b^Adjusted odds ratio excludes respondents from Ghent, NOSM and SHS

^c^Classification of regionally-based medical schools excluded NOSM and SHS on the grounds of insufficient sample size, and excluded Ghent due to differing concepts of rurality

### Students from underserved populations

Those respondents who self-identified as coming from an underserved sociocultural group at entry showed a positive intention towards rural practice location (χ^2^ = 64.5, df = 1, *p* < 0.001), compared with those from the majority socio-cultural group. The underserved sociocultural group was statistically less likely to report intending to work with Indigenous populations (χ^2^ = 5.3, df = 1, *p* = 0.02). Interestingly, an intention to work with migrant or refugee populations at both entry and exit was more likely for respondents that did not report coming from an underserved group (χ^2^ = 9.4, df = 1, *p* = 0.002 and χ^2^ = 5.2, df = 1, *p* = 0.02, respectively).

### Intention to work with underserved populations at entry and exit

At entry to the medical program, 77.5% (1885/2433) of survey respondents indicated that they were “very likely” or “extremely likely” to work with at least one underserved population (rural/remote, Indigenous, mentally ill, immigrant, urban disadvantaged). Acknowledging different cohorts of students, at exit from medical school the corresponding factor for intent to work with underserved populations was similar at 77.4% (595/769; OR 1.01, 95% CI 0.83–1.22, *p* = 0.95).

### Intention to work abroad

Considering respondents from all schools, intention to work abroad at entry (1035/1599, 64.7%) was significantly higher than at exit from medical school (262/493, 53.1%, OR 1.62, *p* < 0.001). There was also a significant inverse association between rural background and intention to practice abroad at entry (rural 339/621, 54.6% vs urban 489/744, 65.7%, χ^2^ = 17.6, df = 1, *p* < 0.001), but not at exit. However, after adjusting for socio-demographic factors, respondents from an urban background were 1.4 times more likely to intend to work abroad (1.41, 95% CI 1.05–1.88, *p* = 0.02; Table 6). Parental family income was also associated with intent to practice abroad with respondents from the top two quintiles more likely to state an intention to work abroad at both entry (264/355, 74.4% vs 377/697, 54.1%, χ^2^ = 40.6, df = 1, *p* < 0.001) and exit (109/174, 62.6% vs 92/224, 41.1% χ^2^ = 18.2, df = 1, p < 0.001). These associations remained significant even after controlling for respondent’s age, gender, income, identifying as a member of an underserved group and geographic background (Table 6).

**Table 6:** Predictors of intention to work abroad where binary variable is “yes – intend to work abroad” and “No – don’t intend to work abroad”. (Unsure option removed from analysis)

	Number in unadjusted analysis	Unadjusted odds ratios (95% CI; *p*-value)	Adjusted odds ratios (95% CI; *p*-value) (*n* = 935)
Age	1829	0.91 (0.89–0.93; < 0.001)	0.89 (0.85–0.92; < 0.001)
Female	1853	1.12 (0.92–1.36; 0.251)	1.03 (0.77–1.37; 0.848)
Income top two quintiles	1223	2.83 (2.19–3.67; < 0.001)	2.08 (1.52–2.85; < 0.001)
Does not identify as underserved group	1669	3.03 (2.42–3.80; < 0.001)	1.96 (1.42–2.72; < 0.001)
Urban background (Quintiles 4 and 5)	1571	1.44 (1.18–1.77; < 0.001)	1.41 (1.05–1.88; 0.020)

Excludes respondents from Ghent University

When considering intention to work abroad, an additional analysis was performed including only the two African and the two Philippines schools, for whom medical emigration is a major concern. At entry, 59.6% (518/869) participants from the Philippines and African schools intended to practice abroad after graduation. There was a statistically significant association between urban background and intention to emigrate (χ^2^ = 20.7, df = 1, *p* < 0.001). Furthermore, 76.6% (82/107) participants from the upper two quintiles for income intended to work abroad compared with 50.7% (232/458) participants from lower three quintiles (χ^2^ = 23.7, df = 1, *p* < 0.001). At exit from medical school, only 37.0% (67/181) of participants from schools in the Philippines and Africa intended to practice abroad; a significantly lower percentage (OR 2.51, *p* < 0.001) than at entry. These significant associations were maintained when adjusted for age, gender, identifying as a member of an underserved group and geographic background (Table 7).

**Table 7:** Predictors of intention to work abroad at African and Filipino schools where binary variable is “yes – intend to work abroad” and “No – don’t intend to work abroad”. (Unsure option removed from analysis)

	Number in unadjusted analysis	Unadjusted odds ratios (95% CI; *p*-value)	Adjusted odds ratios (95% CI; *p*-value) (*n* = 591)
Age	1037	0.83 (0.80–0.86; < 0.001)	0.82 (0.76–0.87; < 0.001)
Female	1048	0.97 (0.76–1.24; 0.818)	1.07 (0.74–1.55; 0.72)
Income top two quintiles	709	3.39 (2.22–5.18; < 0.001)	2.49 (1.50–4.13; < 0.001)
Does not identify as underserved group	970	2.55 (1.95–3.32; < 0.001)	1.66 (1.12–2.46; 0.012)
Urban background (Quintiles 4 and 5)	942	1.73 (1.34–2.24; < 0.001)	1.82 (1.23–2.68; 0.002)

### Intended practice discipline

**Fig. 1:**
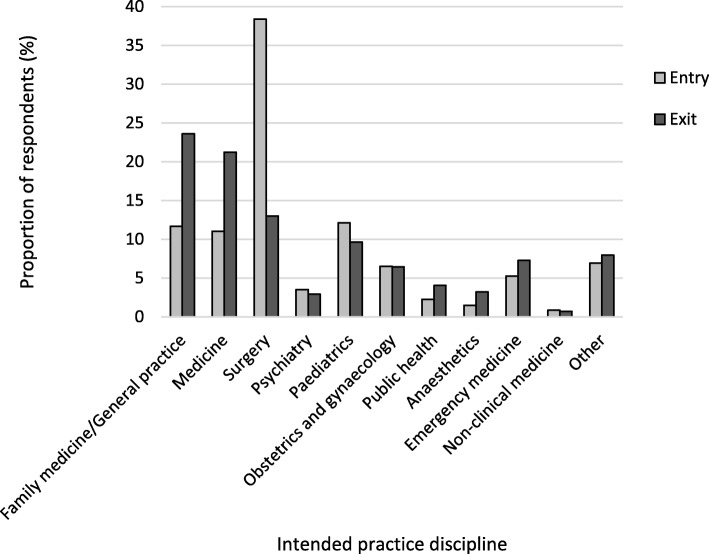
Intended practice discipline of respondents for entry and exit cohorts as a proportion of total respondents who answered this question (Entry *n* = 1766, Exit *n* = 716; Excludes response option ‘I don’t know’)

At exit, the percentage of respondents who intended to practice in family medicine/general practice (169/716, 23.6%) was double that at entry (206/1766, 11.7%, Odds ratio 2.34, 95% CI 1.87–2.93, *p* < 0.001). There was a similar doubling (although smaller in absolute numbers) for intention to practice in public health, comparing respondents at exit with those at entry (Fig. 1). In contrast, fewer respondents at exit (93/716, 13.0%) intended to practice in surgery compared with respondents at entry (678/1766, 38.4%, OR 0.24, 95% CI 0.9–0.30, *p* < 0.001).

## Discussion

This study is constructing an international longitudinal cohort of medical students and graduates from a network of schools in varied contexts attempting to address the mal-distribution of the medical workforce through socially accountable medical education. Although comparison data are limited, from the data collected to date it seems clear that larger proportions of student respondents at THEnet schools come from the lowest two socio-economic quintiles, from non-urban backgrounds and from underserved population groups when compared with other medical school students [19]. Given that these quintiles are derived from population norms, as such, respondents at THEnet schools are likely to more closely reflect population demographics.

The lower proportion of students self-reporting that they come from a minority population group at exit relative to entry is of concern. If this association, demonstrated with cross-sectional data from different cohorts of students, persists into the longitudinal component, it may reflect challenges to retention of these students. Many of these students from underserved groups are first in their family to go to university and have disadvantaged academic backgrounds, [20, 21] though the evidence in the literature is inconclusive [22]. Although THEnet schools are successful at recruiting and admitting a broad cross-section of students, university support structures remain perhaps insufficient for the diversity of their needs; this is an area for further work.

In this study, respondents from economically disadvantaged and rural backgrounds demonstrate a higher intent to work with these populations and in underserved areas in their future practice, both at entry to and exit from medical school. Overall, intention to practice in underserved areas was strongly associated with both rurality of background and parental income but also with a regional location of the medical school.

Importantly, although intention to practice in rural areas was slightly lower in exit compared to entry cohorts, overall intention to practice with underserved populations did not differ between entry and exit cohorts at these THEnet schools. This is despite evidence from traditional schools that altruism and a desire to serve in areas of need usually wanes through medical education in response to urban tertiary hospital environments and a pervasive hidden curriculum [23, 24]. Other related constructs that might influence intended and actual practice in underserved areas include a sense of obligation or an implicit social contract. In some THEnet schools (for example, University of the Philippines School of Health Sciences-Leyte), this contract is explicit, with communities sponsoring a young person to study a health career with an expectation that they will return and provide service in the community. We will be collecting prospective practice data from all schools (both during and beyond return of service periods) as part of this study to help understand the contribution of any real or altruistic service obligations.

For low and middle income countries, retaining their trained health workforce and minimizing emigration is a critical outcome. For the schools in the Philippines and Africa where this is most relevant, there was a clear inverse association between socioeconomic background and intention to emigrate, and importantly, the intention to emigrate from students at these schools was lower in students at exit compared to entry, suggesting that they are being socialised towards service.

There is of course a considerable gap between practice intentions and actual practice. However, as outlined below, published studies of graduating cohorts from THEnet schools James Cook University (JCU), Ateneo de Zamboanga University School of Medicine (ADZU) and the Northern Ontario School of Medicine (NOSM), using slightly varying methodologies, suggest that actual practice in underserved areas is likely to be substantial. For example, published outcome figures for the first seven cohorts of graduates from the JCU medical program in northern Australia demonstrate extremely high retention in rural and remote Australia, with 44% of graduates completing their internship in outer regional and rural areas (Australian Standard Geographical Classification 3–5) and 64% completing their internship outside capital cities (ASGC 2–5) [25]. Furthermore, 34% of known graduates between PGY4 and PGY9 pursued graduate careers in Northern Australia, [26] with 39% of graduates between 2005 and 2008 practicing in outer-regional to remote locations (ASGC3–5) at PGY5 [27]. Similarly, 61% of the first three cohorts of family practitioners graduating from NOSM practised in the underserved region of Northern Ontario in 2014, with the percentage rising to 94% for those who completed both their undergraduate and postgraduate medical education at NOSM [28]. In a low income setting, ADZU has demonstrated results in terms of the number of graduates remaining in Mindanao, an underserved part of the southern Philippines. In contrast to other medical schools in the Philippines, where up to 80% of graduates emigrate, 90% of ADZU graduates remain working in the southern Philippines where they are needed [6].

Further qualitative work is exploring changes in student values and desire for service over the medical education program at THEnet schools [29] and the role of faculty motivation and faculty background in contributing to these outcomes.

### Strengths and limitations

This is the largest international survey of medical student practice intentions available, and involves medical schools from the most diverse contexts. Collecting data of this kind across schools and contexts is challenging, and there is some inherent messiness involved [30]. As we are building up a longitudinal cohort study across countries, there is a time lag of between 4 and 6 years from when the students enter and graduate from medical school and thus when linked longitudinal data become available. Thus, we have elected to report interim findings from this sizable international database using two sets of cross-sectional data, recognising inherent limitations, including some significant differences in parental family incomes, parental education or belonging to an underserved population between entry and exit groups—differences that could have affected our results. In future, when we have increasing follow up of graduating cohorts, then we expect to report more broadly on the degree to which practice intentions translate into actual practice (clearly the more important measure).

There are complexities associated with this type of research across continents and among schools that differ in the resources available for data collection and management. Strengthening capacity for ongoing evaluation of the outcomes and impact of schools in under-resourced settings is an important facet of this work. In addition, some schools are already reporting on difficulties with following up graduates after they leave the school – this varies according to the strength of alumni networks, the existence of strong regulatory bodies and professional organisations and the involvement of the school in specialty training programs.

Our method of agreeing on a definition of underserved groups across contexts is imperfect, but the best compromise possible allowing comparability [12]. There are also issues with comparing quintiles of rurality and of family socioeconomic status, derived at individual school level. In particular, we recognize that rurality measures based on population size are imperfect, failing to take into account travel time and accessibility to regional centres – some, but not all, schools were able to account for this. Despite these limitations, this large and growing database is an important resource in understanding how health professional education can meet current and future population health care needs.

### Implications

These findings suggest that medical education with a focus on social accountability is likely to produce a medical workforce with strong intentions to work with underserved populations in their regions. However, an aligned workforce presents major challenges for medical schools in terms of broadening admission policies beyond purely academic achievement and then providing adequate academic, social and pastoral support for students from non-traditional academic backgrounds. Furthermore, it is important to consider the impact of the cost of applying to medical school and the cost of the schooling – these might be prohibitive for students from low socioeconomic status backgrounds; the very students who are most likely to remain in the country and serve where the needs are the greatest.

## Conclusion

These data confirm that rurality of origin and other measures of disadvantage predict intention to work with underserved populations across eight medical schools in five continents. Furthermore, intention to practice with underserved communities is similar or higher upon exit from the program. These cross-sectional findings suggest that the student respondents’ experience throughout the program is training and socializing graduates towards meeting population need. Although further research is necessary to better understand how various factors intersect to produce these results, it seems clear that these medical schools with social accountability missions are delivering on their promises. Our results suggest that broadening the population base from which medical students were historically selected and training them in regional and rural areas will facilitate the production of a medical workforce that is skilled and distributed according to health care need.
